# Supplementary material for: The Influence of Flower Head Order and Gibberellic Acid Treatment on the Hydroxycinnamic Acid and Luteolin Derivatives Content in Globe Artichoke Cultivars
Source: Foods. 2021 Aug 5;10(8):1813. doi: 10.3390/foods10081813 (PMC8394965; doi:10.3390/foods10081813)
Supplement: Supplementary file 1 [file foods-10-01813-s001.zip › foods-1273953-supplementary.pdf]

The influence of flower head orders and gibberellic acid treatment on the hydroxycinnamic acid and luteolin derivatives content in globe artichoke cultivars

María J. Giménez[1], Marina Giménez-Berenguer[1], María E. García-Pastor[1], Joaquín Parra[2], Pedro J. Zapata[1] and Salvador Castillo[1,*]

[1] Department of Food Technology, EPSO, University Miguel Hernández, Ctra. Beniel km. 3.2, 03312 Orihuela, Alicante, Spain; maria.gimenezt@umh.es (M.J.G.); marina.gimenez02@goumh.umh.es (M.G.-B.); m.garciap@umh.es (M.E.G.-P.); pedrojzapata@umh.es (P.J.Z.)
[2] Elche Agricultural Experimental Station (EEA/STT), CV-855, Ctra. Dolores km. 1, 03290 Elche, Alicante, Spain; parra_joa@gva.es (J.P.)
* Correspondence: scastillo@umh.es; Tel.: +34-966-749-733

Supplementary Material

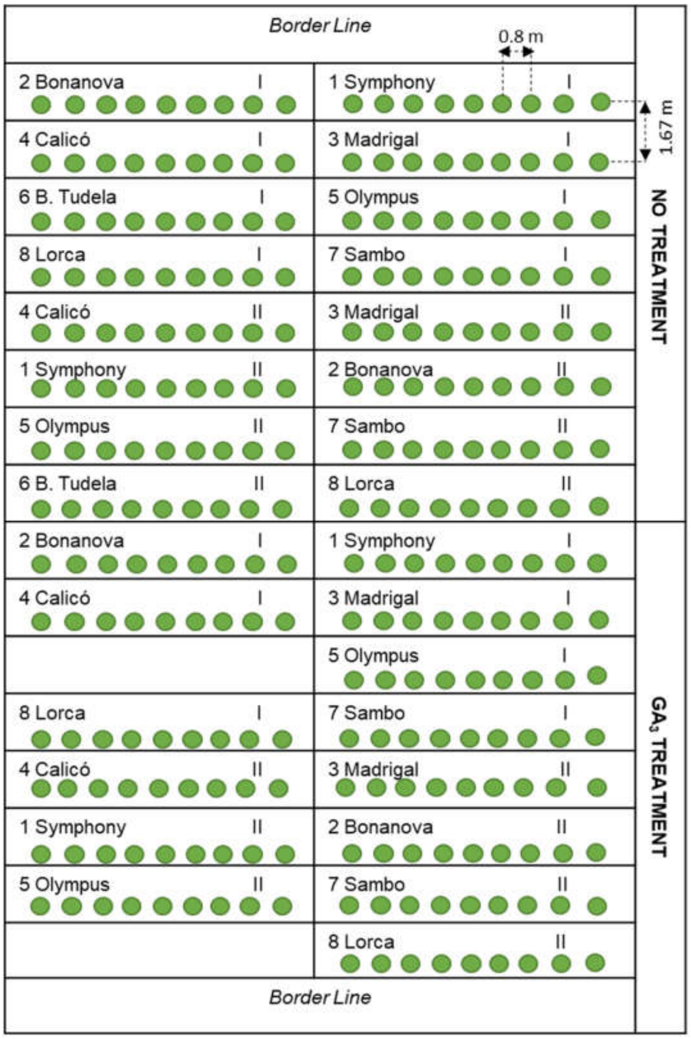

Figure S1. Plot experimental design of the two assayed blocks (block I and II) for untreated artichokes (no treatment) and artichokes treated with gibberellic acid (GA_3) in the eight cultivars.

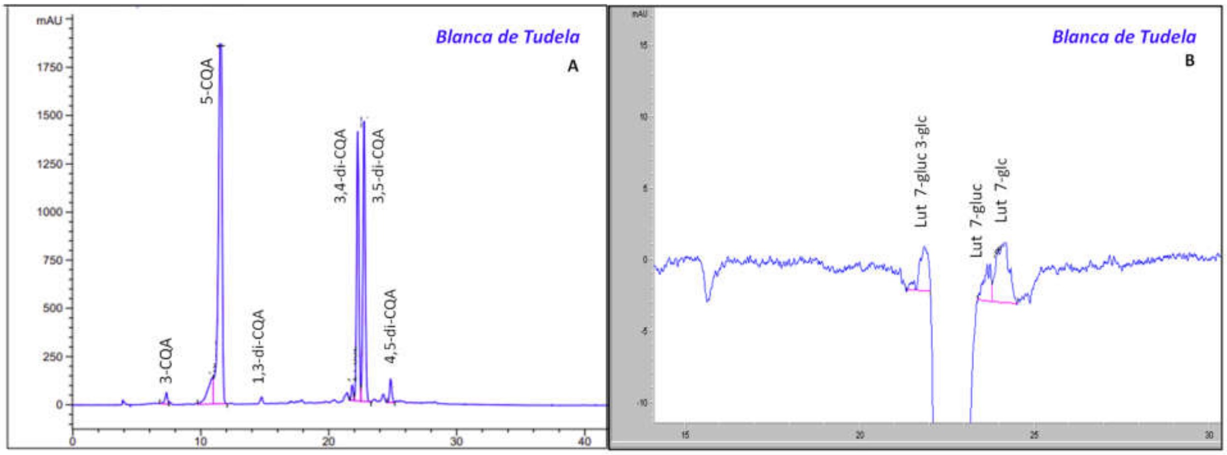

Figure S2. HPLC-DA-ESI/MS^n chromatogram at 320 nm for the 'Blanca de Tudela' cultivar **(A)**. The hydroxycinnamic acids identified: 3-CQA (3-*O*-caffeoylquinic acid; $t_r \approx 7.3$ min); 5-CQA (5-*O*-caffeoylquinic acid; $t_r \approx 11.5$ min); 1,3-diCQA (1,3-di-*O*-caffeoylquinic acid; $t_r \approx 14.7$ min); 3,4-diCQA (3,4-di-*O*-caffeoylquinic acid; $t_r \approx 22.2$ min); 3,5-diCQA (3,5-di-*O*-caffeoylquinic acid; $t_r \approx 22.7$ min); and 4,5-diCQA (4,5-di-*O*-caffeoylquinic acid; $t_r \approx 24.8$ min). RP-HPLC-DAD chromatogram at 360 nm for the 'Blanca de Tudela' cultivar **(B)**. Luteolin derivatives identified: luteolin 7-*O*-glucuronide 3-*O*-glucoside (Lut 7-gluc 3-glc; $t_r \approx 21.9$ min); luteolin 7-*O*-glucuronide (Lut 7-gluc; $t_r \approx 23.5$ min); and luteolin 7-*O*-glucoside (Lut 7-glc; $t_r \approx 23.8$ min).

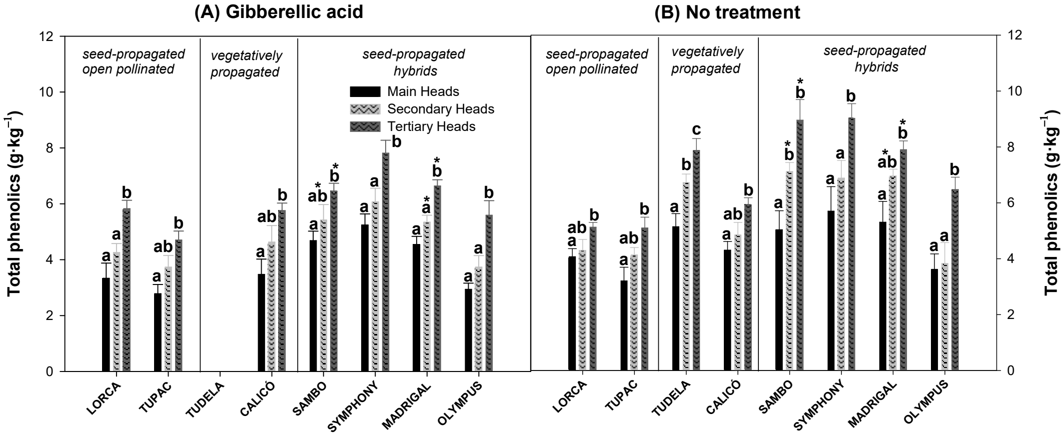

Figure S3. Total phenolic content (g·kg^{-1}) of globe artichoke cultivars influenced by flower head order (main, secondary and tertiary heads) and treatment [gibberellic acid **(A)** or no treatment **(B)**]. Data are the mean ± SE. Different letters show significant differences ($p < 0.05$ according to HSD Duncan's test) among flower head orders for each artichoke cultivar and treatment. Significant differences between both treatments ($p < 0.05$ according to Student's t-test) were expressed as * placed in each flower head order for each artichoke cultivar.

Table S1. Heatmap's supplementary data of minor hydroxycinnamic acid content (Figure 5): 3-*O*-caffeoylquinnic acid (3-CQA), 1,3-di-*O*-caffeoylquinnic acid (1,3-diCQA) and 4,5-di-*O*-caffeoylquinnic acid (4,5-diCQA) content ($mg \cdot kg^{-1}$) of globe artichoke cultivars influenced by flower head order (main, secondary and tertiary heads represented in the heatmap with the numbers 1, 2 and 3) and treatment (gibberellic acid or no treatment).

		**Gibberellic acid**			**No treatment**		
		**Main heads**	**Secondary heads**	**Tertiary heads**	**Main heads**	**Secondary heads**	**Tertiary heads**
**3-*O*-caffeoylquinic acid**	**LORCA**	206.9 ± 18.3a	292.4 ± 10.8b*	303.4 ± 16.7b	179.7 ± 4.9a	223.1 ± 0.3b*	242.3 ± 21.2b
	**TUPAC**	119.9 ± 10.8a*	222.1 ± 18.0b*	301.8 ± 19.7c*	157.2 ± 7.9a*	168.4 ± 8.7a*	183.4 ± 12.5a*
	**TUDELA**	-	-	-	23.9 ± 1.3a	29.2 ± 1.5b	45.5 ± 4.2c
	**CALICÓ**	84.4 ± 6.5a	89.7 ± 7.7a	113.7 ± 10.5a	89.7 ± 7.7a	106.2 ± 4.8ab	132.0 ± 12.5b
	**SAMBO**	57.5 ± 5.0a	57.9 ± 4.2a*	67.0 ± 5.2a*	67.9 ± 4.7a	95.9 ± 5.0b*	96.0 ± 8.5b*
	**SYMPHONY**	151.1 ± 6.1a*	175.3 ± 10.1ab*	222.2 ± 19.1b*	90.1 ± 7.1a*	117.0 ± 6.8b*	134.7 ± 7.3b*
	**MADRIGAL**	86.4 ± 7.2a	116.5 ± 9.3b	126.2 ± 6.8b*	65.0 ± 4.5a	93.5 ± 7.6b	96.9 ± 6.4b*
	**OLYMPUS**	120.3 ± 8.3a*	179.5 ± 11.0b	232.6 ± 19.6b	154.9 ± 1.6a*	178.0 ± 8.7b	219.9 ± 7.6c
**1,3-*O*-dicaffeoylquinic acid**	**LORCA**	2.3 ± 0.2a*	2.4 ± 0.2a*	14.4 ± 0.8b*	14.9 ± 0.7a*	22.4 ± 1.9b*	28.5 ± 1.9b*
	**TUPAC**	2.6 ± 0.2a*	4.8 ± 0.4b*	4.9 ± 0.4b*	16.4 ± 0.9a*	18.3 ± 1.1a*	37.3 ± 2.2b*
	**TUDELA**	-	-	-	23.1 ± 0.7a	27.7 ± 1.4b	29.0 ± 1.1b
	**CALICÓ**	18.9 ± 1.6a*	21.4 ± 1.1a	23.5 ± 1.8a	21.4 ± 1.1a*	25.8 ± 1.7b	27.7 ± 1.5b
	**SAMBO**	4.7 ± 0.3a*	7.4 ± 0.2b*	12.1 ± 1.0c*	44.8 ± 4.1a*	47.4 ± 1.7a*	52.3 ± 4.7a*
	**SYMPHONY**	5.6 ± 0.3a*	7.3 ± 0.5b*	9.2 ± 0.8b*	25.3 ± 1.8a*	48.7 ± 4.5b*	46.7 ± 3.4b*
	**MADRIGAL**	6.1 ± 0.4a*	9.5 ± 0.8b*	11.4 ± 1.0b*	47.0 ± 3.4a*	47.8 ± 3.3a*	70.3 ± 5.5b*

	**OLYMPUS**	4.5 ± 0.4a*	4.5 ± 0.3a*	5.0 ± 0.5a*	22.5 ± 0.19a*	25.1 ± 1.9ab*	30.1 ± 1.0b*
**4,5-*O*-dicaffeoylquinic acid**	**LORCA**	40.7 ± 2.6a	42.2 ± 2.8a	59.0 ± 4.4b	33.5 ± 2.2a	46.4 ± 2.7b	54.0 ± 6.3b
	**TUPAC**	57.6 ± 3.2a*	47.9 ± 2.2a*	33.9 ± 1.6b*	28.8 ± 1.8a*	38.0 ± 2.7b*	50.0 ± 3.3c*
	**TUDELA**	-	-	-	70.4 ± 5.8a	89.1 ± 3.8b	95.9 ± 6.9c
	**CALICÓ**	62.0 ± 3.6a*	63.8 ± 4.0a*	68.7 ± 2.8a*	100.0 ± 3.6a*	108.7 ± 3.6a*	214.2 ± 16.2b*
	**SAMBO**	79.3 ± 7.6a	89.1 ± 2.5a	92.9 ± 4.7a*	83.7 ± 4.1a	95.4 ± 3.0a	191.4 ± 22.3b*
	**SYMPHONY**	85.6 ± 2.5a	90.9 ± 8.5a	104.9 ± 9.5a*	84.9 ± 5.7a	86.3 ± 6.9a	225.5 ± 10.6b*
	**MADRIGAL**	95.3 ± 2.6a*	103.3 ± 9.1a*	150.4 ± 10.3b*	138.6 ± 12.4a*	155.9 ± 12.8a*	314.5 ± 7.8b*
	**OLYMPUS**	37.4 ± 2.6a	73.8 ± 1.7b*	128.3 ± 11.2c*	39.8 ± 2.3a	51.0 ± 2.4b*	52.3 ± 2.9b*

[1] Different letters, within the same row, show significant differences ($p < 0.05$ according to HSD Duncan's test) among flower head orders for each artichoke cultivar and treatment. Significant differences between both treatments ($p < 0.05$ according to Student's t-test) were expressed as * placed in each flower head order for each artichoke cultivar.
